# Mitochondrial DNA as an inflammatory mediator in cardiovascular diseases

**DOI:** 10.1042/BCJ20170714

**Published:** 2018-03-06

**Authors:** Hiroyuki Nakayama, Kinya Otsu

**Affiliations:** 1 Laboratory of Clinical Science and Biomedicine, Graduate School of Pharmaceutical Sciences, Osaka University, Suita, Osaka 565-0871, Japan; 2 The School of Cardiovascular Medicine and Sciences, King's College London British Heart Foundation Centre of Research Excellence, London SE5 9NU, U.K.

**Keywords:** cardiovascular disease, inflammation, mtDNA

## Abstract

Mitochondria play a central role in multiple cellular functions, including energy production, calcium homeostasis, and cell death. Currently, growing evidence indicates the vital roles of mitochondria in triggering and maintaining inflammation. Chronic inflammation without microbial infection — termed sterile inflammation — is strongly involved in the development of heart failure. Sterile inflammation is triggered by the activation of pattern recognition receptors (PRRs) that sense endogenous ligands called damage-associated molecular patterns (DAMPs). Mitochondria release multiple DAMPs including mitochondrial DNA, peptides, and lipids, which induce inflammation via the stimulation of multiple PRRs. Among the mitochondrial DAMPs, mitochondrial DNA (mtDNA) is currently highlighted as the DAMP that mediates the activation of multiple PRRs, including Toll-like receptor 9, Nod-like receptors, and cyclic GMP–AMP synthetase/stimulator of interferon gene pathways. These PRR signalling pathways, in turn, lead to the activation of nuclear factor-κB and interferon regulatory factor, which enhances the transcriptional activity of inflammatory cytokines and interferons, and induces the recruitment of inflammatory cells. As the heart is an organ comprising abundant mitochondria for its ATP consumption (needed to maintain constant cyclic contraction and relaxation), the generation of massive amounts of mitochondrial radical oxygen species and mitochondrial DAMPs are predicted to occur and promote cardiac inflammation. Here, we will focus on the role of mtDNA in cardiac inflammation and review the mechanism and pathological significance of mtDNA-induced inflammatory responses in cardiac diseases.

## Introduction

Mitochondria are intracellular double membrane-bound organelles that play central roles in many essential cellular functions, including energy production, calcium homeostasis, and programmed cell death. In the last decade, the additional role and molecular mechanism of mitochondria in antibacterial and antiviral defence as well as inflammation have been revealed [1,2]. Indeed, mitochondria contribute to the innate immune response through the activation of several pathways [3]. The innate immune response provides rapid detection of and protection against microorganisms such as bacteria, virus, and fungi, by sensing pathogen-associated molecular patterns (PAMPs). Pattern recognition receptors (PRRs), including Toll-like receptors (TLRs) and Nod-like receptors (NLRs), sense these PAMPs as well as a wide range of damage-associated molecular patterns (DAMPs). Among them, sensing of pathogen-derived nucleic acids is one of the major mechanisms for innate immune cell activation.

Mitochondria generate and release multiple DAMPs to stimulate the innate immune system through multiple routes and are implicated in a growing list of inflammation-related diseases and pathogeneses. Owing to their bacterial ancestry, mitochondrial DAMPs can bind and activate multiple PRRs similar to those recognized by PAMPs [4]. Among the molecules listed as mitochondrial DAMPs, *N*-formyl peptides, cardiolipin, and mitochondrial DNA (mtDNA) are liberated from mitochondria into the cytosol or the extracellular space in response to cellular stress or after cell death and can activate sterile inflammation [5,6]. Inflammatory responses induced by sterile stimuli are similar to responses during infection, including the recruitment of neutrophils and macrophages (MΦs), production of inflammatory cytokines and chemokines, and induction of T-cell-mediated adaptive immune responses [7]. Of note, mtDNA has recently been established as an important DAMP and a possible trigger of various inflammatory or degenerative diseases [8,9].

Growing evidence indicates that inflammation without microorganisms is strongly involved in the pathogenesis of cardiac diseases [10–13]. As a consequence, cardiac inflammation is proposed to be an important therapeutic target for the treatment of cardiovascular diseases, including ischaemic heart diseases, heart failure, and atherosclerosis [14,15]. For this purpose, the role of mitochondrial DAMPs in cardiac inflammation needs to be clarified, as cardiomyocytes maintain abundant mitochondria. Here, we review the current knowledge and evidence about the pathological roles of mtDNA in cardiac inflammation.

## What is cardiac inflammation?

Inflammation consists of several processes. The first step is the detection of exogenous or endogenous ligands, which trigger the inflammatory response, by PRRs. In general, exogenous ligands often arise from microorganisms such as bacteria, viruses, or chemical substances carrying PAMPs. Endogenous ligands involve intracellular molecules such as nucleotides, proteins, and lipids, which are released after necrotic cell death or stress. The second step is the activation of the signalling of downstream PRRs, which leads to up-regulation of the transcription of genes of inflammatory cytokines, chemokines, and vasoactive amines followed by the extracellular release of those molecules. The third step is the recruitment of professional immune cells including neutrophils, which injure microorganisms through the accumulation of molecules including proteases and reactive oxygen species (ROS) or radical nitrogen species. Recruited macrophages also play a central role in the clearance of ligands or damaged tissues.

In general, inflammation of the heart is divided into two pathologic conditions: inflammatory cardiomyopathies such as myocarditis and cardiac inflammation. Myocarditis triggered by microorganisms such as viruses (adenovirus and enteroviruses) and protozoa (Chagas disease) is associated with massive inflammatory responses and often causes cardiac dysfunction. Autoimmunity is also involved in diseases such as sarcoidosis and autoimmune myocarditis. These diseases are categorized as inflammatory cardiomyopathies and were recently highlighted in an excellent review by Trachtenberg and Hare [16]; thus, we will not describe them here. Cardiac inflammation, which is the main focus of this review, indicates non-infectious inflammation or what is called ‘sterile inflammation’, which frequently occurs as a secondary response associated with myocardial damage from ischaemia or other causes of heart failure.

Multiple reports have indicated that inflammation plays a significant role in the development of heart failure [17]. The levels of inflammatory cytokines (tumour necrosis factor-α [TNF-α], interleukin (IL)-1β, and IL-6) are increased in patients with heart failure [18]. Increased levels of these cytokines and their receptors are independent risk factors of mortality in patients with advanced heart failure [19], or of poor prognosis in patients with idiopathic dilated cardiomyopathy [20]. Moreover, a significant correlation between the serum levels of TNF-α and the severity of heart failure has been reported [18].

Mechanistically, cytokines such as TNF-α and IL-1β mediate the down-regulation of Ca^2+^-cycling-associated genes such as sarcoplasmic reticulum Ca^2+^ ATPase (*SERCA2*) via activation of nuclear factor kappa B (NF-κB) [21], which leads to reduction in contractility through alterations in intracellular Ca^2+^ homeostasis in adult cardiac myocytes [22–24]. This inflammation-triggered disturbance of Ca^2+^ homeostasis in cardiomyocytes is possibly involved in the process of cardiac remodelling, generating a vicious circle [25]. In addition, TNF-α and IL-1β induce cardiomyocyte hypertrophy [26], which is another independent risk factor of heart failure [27]. TNF-α also triggers cardiomyocyte apoptosis [28], which eventually results in considerable myocyte loss leading to the development of heart failure. Alternatively, IL-6 has been reported to increase cardiomyocyte stiffness through the reduction of titin phosphorylation [29].

There are a variety of sources of cytokine production in the heart, including almost all cardiac cells such as cardiomyocytes, endothelial cells, cardiac fibroblasts, and resident macrophages [30–33]. Moreover, the secreted cytokines induce the infiltration of extra-cardiac immune cells such as neutrophils and macrophages, which produce cytokines/chemokines during pathological conditions. A vicious circle is generated, resulting in chronic inflammation in the heart [34]; however, the precise mechanisms of the initial trigger and of the maintenance of chronic inflammation are still elusive.

Transforming growth factor (TGF)-β mediates the trans-differentiation of fibroblasts to active myofibroblasts. Myofibroblasts exhibit enhanced production of collagens and inflammatory cytokines compared with quiescent fibroblasts [35]. In addition, activated myofibroblasts elicit cardiomyocyte hypertrophy and dysfunction through the secretion of pro-hypertrophic inducers, including Ang II (angiotensin II), TGF-β1, and fibroblast growth factor [36,37]. Myofibroblasts also stimulate monocytes to express gelatinases, which enhance the permeability of the microvasculature; induce subsequent infiltration of immune cells in the heart [34,35]; and modulate the polarity of macrophages [38]. On the other hand, cardiac endothelial cells [31] are important sources of IL-1β, one of the end products of NLRP3 (NLR family pyrin domain containing 3) inflammasome activation.

Besides endogenous cardiac cells, infiltrated immune cells are responsible for the regulation of inflammation. Activated neutrophils produce large amounts of ROS, which are critical in host defence and cause tissue damage [39]. The M2 macrophage plays a significant role in resolving inflammation by removing dead cells through phagocytosis.

To date, despite extensive evidence from clinical and basic research, no therapeutic approaches with anti-inflammatory drugs have demonstrated beneficial effects in clinical trials. Indeed, disappointing results of anti-inflammatory strategies have been shown in double-blind clinical trials targeting TNF-α in patients with heart failure [40]. These results indicate that optimized anti-inflammatory strategies are required to establish novel therapeutics, as inflammation itself is diverse and complex.

## Mitochondrial DAMPs and sterile inflammation

### mtDNA and inflammation

mtDNA is a small, double-stranded circular molecule, encoding 13 respiratory chain polypeptides, together with transfer and ribosomal RNAs that are needed for their translation in the mitochondrial matrix [41]. In humans, mtDNA exists as a 16 569-bp loop, and polymerase gamma is the mtDNA polymerase uniquely responsible for replicating the mitochondrial genome [42]. There are ∼1200 mitochondrial proteins that are encoded in the nuclear genome and imported into the organelles, and they function in the expression and maintenance of mtDNA [43,44]. Transcriptional co-activators, including the peroxisome proliferator-activated receptor gamma co-activator-1 family, nuclear respiratory factors 1 and 2, and oestrogen-related receptor α, orchestrate the expression of those mitochondrial proteins [45]. There are specific mitochondrial proteins that bind to mtDNA and form a complex called nucleoids [44]. The nucleoid is an area in the mitochondrion that contains DNA associated with proteins necessary for the maintenance of mtDNA integrity. The mtDNA-binding protein transcription factors A (TFAM), B1 (TFB1M), and B2 (TFB2M) are encoded in the nuclear genome, and once expressed are then transported into mitochondria by a protein import machinery. TFAMs belong to the high-mobility group proteins and associate with the inner mitochondrial membrane. TFAMs can form nucleoids by binding mtDNA without sequence specificity, and participate in mtDNA transcription and replication [46,47].

mtDNA has unique features that are different from those of the nuclear genome. First, similar to the bacterial genome, mtDNA contains a predominantly unmethylated CpG motif, although the precise degree of CpG methylation has yet to be determined [47,48]. Second, it has been considered that mtDNA is prone to damage owing to its lack of packaging by protective histones and its proximity to the sources of mitochondrial ROS (mtROS). Currently, mtDNA is known to be more resistant to damage than expected, owing to its binding to TFAM proteins [49]. Third, mtDNA exhibits inefficient DNA repair mechanisms compared with nuclear DNA, as mitochondria lack nucleotide excision repair, which functions in the nucleus [50–52].

Collins et al. [4] first reported the immunostimulatory potential of mtDNA in 2004. They found that intra-articular injection of mtDNA, but not nuclear DNA, triggered inflammatory arthritis in mice by inducing the secretion of TNF-α in splenocytes. Consistently, depletion of mtDNA attenuated IL-1β production in macrophages through the inhibition of inflammasome activation following treatment with lipopolysaccharide (LPS) and ATP [53]. It is considered that mtDNA mediates inflammation in a similar manner by which bacterial unmethylated CpG exerts inflammation through PRR activation. Noticeably, the pro-inflammatory effects of mtDNA are dependent on its oxidization [54,55]. TFAM binding of mtDNA confers nucleotide stability and, when unbound, mtDNA becomes more fragile and prone to degradation. Oxidative modifications occurring at the level of TFAM or mtDNA are indicated as major elements affecting TFAM binding and resulting in nucleoid instability [56]. However, whether defective TFAM binding to mtDNA is responsible for the activation of inflammatory responses remains unclear. Both cell-free mtDNA and TFAM-bound mtDNA are reported to induce a systemic inflammatory response [56].

### Sensors for detecting mtDNA

**Figure 1.**
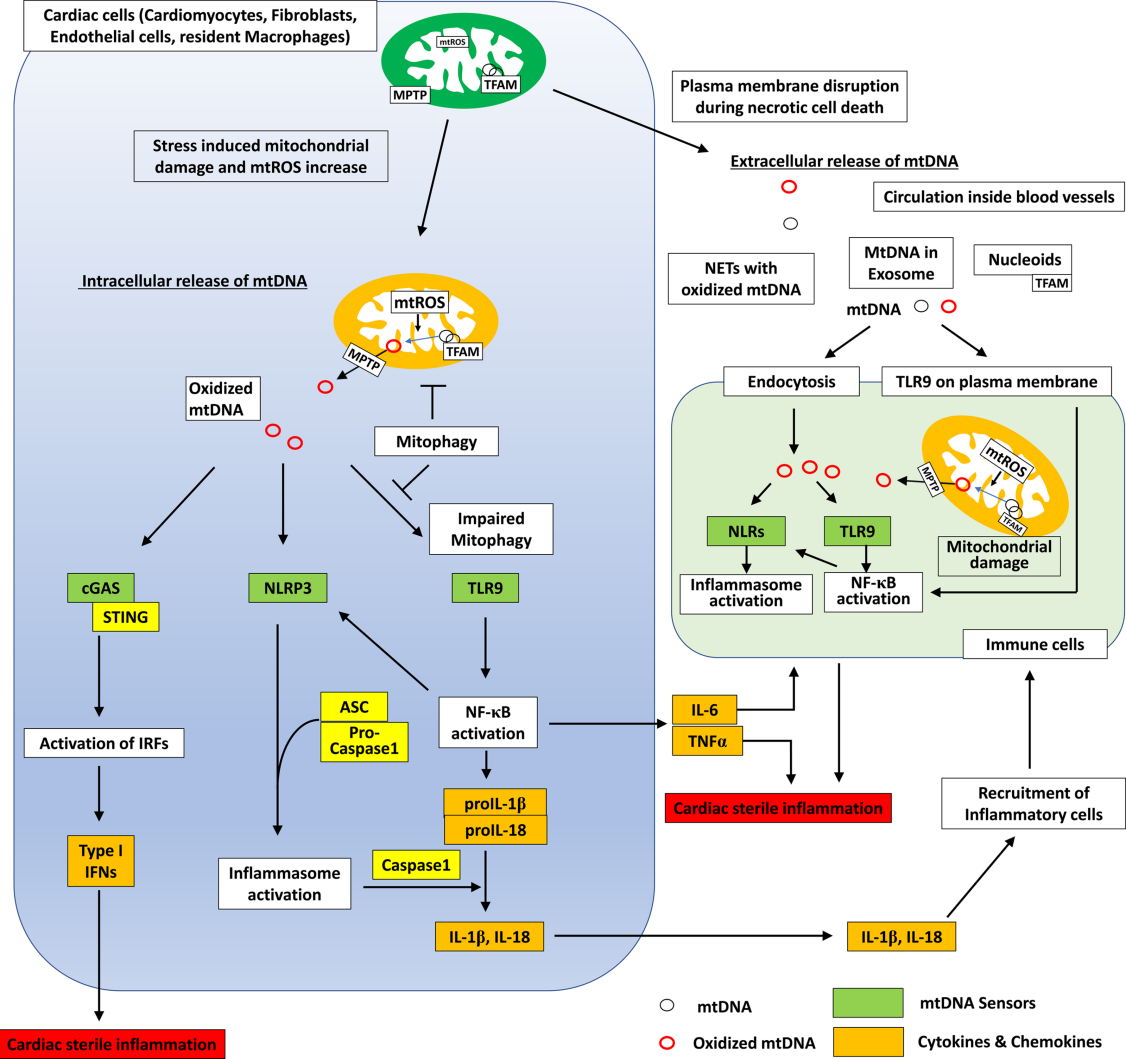
Mitochondrial DNA and cardiac inflammation. mtDNA binds to TFAM and is stabilized in cardiac cells, including cardiomyocytes, cardiac fibroblasts, and endothelial cells. Increase of mtROS during stress stimulation leads to oxidation of mtDNA and dissociation of TFAM. Oxidized mtDNA is released via the mitochondrial permeability transition pore (MPTP), whose opening is regulated by cyclophilin D. Damaged mitochondria are degraded by the autophagic process, mitophagy, and detoxified. When this process is impaired, mtDNA inside the autolysosome escape degradation and stimulate TLR9 to induce NF-κB activation, which causes transcriptional activation of multiple inflammatory cytokines (IL-6, TNF-α, pro-IL-1β, and pro-IL-18). NF-κB activation also enhances transcription of NLRP3 to prime inflammasome activation. Increased NLRP3 senses mtDNA and forms a protein complex called inflammasome with ASC and pro-caspase 1, which finally activates caspase 1 to cleave to pro-IL-1β and pro-IL-18 to transform these molecules into bioactive cytokines. Secreted inflammatory cytokines from cardiac cells mediate recruitment of inflammatory cells and cardiac sterile inflammation. cGAS senses mtDNA and activates interferon-related factors to increase transcriptional activities of type I interferons, which cause cardiac inflammation. On the other hand, extracellular mtDNA is released and circulates inside vessels as cell-free mtDNA when the plasma membrane is disrupted by tissue damage, and necrotic cell death is induced. In the serum, mtDNA is observed within exosomes, TFAM-bound forms (nucleoids), or inside neutrophil extracellular traps (NETs). mtDNA enters the endocytic pathway by endocytosis and stimulates endosomal TLR9, which leads to NF-κB activations and inflammasome formation. These processes can be involved in the development of cardiac sterile inflammation.

Specific characteristics of mtDNA, such as its relative hypomethylation, unique structural features, and susceptibility to oxidative damage owing to its close proximity to massive ROS sources, make it a potential potent DAMP that activates innate immunity to trigger pro-inflammatory processes and type I interferon (IFN) responses. Current evidence indicates that mtDNA-mediated inflammation is caused by the activation of the TLR9, NLRP3 inflammasome, and cyclic GMP–AMP synthetase (cGAS)–STING (stimulator of interferon gene) DNA-sensing pathways (Figure 1) [4,57].

#### Toll-like receptor 9

TLR9 is the endosomal TLR that senses bacterial and viral DNAs, and its ligands can preferentially activate downstream pathways in a Myd88-dependent manner. This pathway finally culminates in the activation of NF-κB, which leads to transcriptional up-regulation of pro-inflammatory cytokines such as IL-6 and pro-IL-1β, as well as NLRP3 and IFN regulatory factor (IRF)-dependent type 1 IFN [58,59]. Currently, TLR9 activation is considered a priming step for NLRP3 inflammasome activation through NF-κB activation and subsequent downstream signalling [60].

#### NLRP3 and inflammasomes

NLRP3 is the second sensor to link redox state and mtDNA with inflammation. NLRP3 senses multiple danger stimuli, including viruses, bacterial toxins, and crystallized cholesterol [61]. The involvement of mtROS in NLRP3 activation has been reported and may be explained by its oxidizing effects on mtDNA [53,54,62,63]. mtROS enhance the cytosolic translocation of oxidized mtDNA, which binds NLRP3 and activates the NLRP3 inflammasome, a multi-protein complex composed of NLRP3, an apoptosis-associated speck-like protein containing a caspase activation and recruitment domain (ASC), and caspase-1 [54]. Once activated, NLRP3 and ASC co-localize at endoplasmic reticulum–mitochondrial clusters in the perinuclear space to induce cleavage and activation of caspase-1 [64]. The activation of caspase-1 leads to cleavage and transduction of pro-IL-1β and pro-IL-18 to their bioactive form, and these may be involved in redox-sensitive inflammatory responses [65]. In addition, genetic deletion of NLRP3 and caspase-1 leads to reduced mtDNA release [53,63]. On the other hand, non-oxidized mtDNA is reported to stimulate IL-1β production through the activation of other inflammasomes such as AIM2 (absent from melanoma 2) [66].

#### Cyclic GMP–AMP synthetase

The cGAS–STING DNA-sensing pathway is an additional component of the innate immune system [67]. STING is an endoplasmic reticulum-anchored cytosolic protein and can be activated to induce an IFN response through a direct association with dsDNA or through cyclic dinucleotides, which can be derived from microbes such as bacteria or viruses [68]. The STING-mediated IFN response can also be induced by intracellular mtDNA [69,70]. cGAS functions as a DNA sensor. Upon binding to mtDNA, cGAS promotes the recruitment of STING protein, which triggers the phosphorylation of the transcription factor IRF-3 through the TANK (TRAF family member-associated NF-κB activator)-binding kinase and activation of NF-κB signalling [71]. Activated IRF-3 mediates the transcription of type I and III IFNs and IFN-stimulated nuclear gene products, which results in mtDNA-induced inflammatory responses. In the physiological setting, systemic injection of oxidized mtDNA increases IFN-stimulated gene expression in the spleen of wild-type but not STING-deficient mice. In addition, STING also plays a vital role in enhanced type I IFN response caused by increased cytosolic mtDNA in TFAM-deficient cells [72]. When TFAM is heterozygously deleted in mouse embryonic fibroblasts, mtDNA is released to the cytosol, and the DNA-sensing cGAS–STING signalling pathway is activated to enhance the expression of type 1 IFNs and other IFN-related genes.

Recently, it was reported that mtDNA-induced inflammatory pathways are closely related to mitochondrial intrinsic apoptotic pathways. The mitochondrial pro-apoptotic proteins Bak and Bax regulate mitochondrial outer membrane permeability transition, which causes the release of both cytochrome *c* and mtDNA. When apoptosis-processing caspases (3, 7, and 9) are inhibited, cytosolic mtDNA elicits type I IFN responses via the activation of cGAS–STING signalling [69,70]. This evidence suggests that apoptotic caspases contribute not only to cell death processing but also to the silent inflammogenic feature of apoptosis, through the inhibition of mtDNA-induced cGAS–STING signalling.

### Dynamics of mtDNA

It was proposed that circulating cell-free mtDNA is a functional link between mitochondrial damage and systemic inflammation [4,56]. Indeed, mtDNA, which is released after cell death, functions as a DAMP and can induce an inflammatory response through hypomethylated CpG motifs resembling those of bacterial DNA [4]. However, there are considerable uncertainties in the process of the activation of the above-mentioned sensors. There are two routes of mtDNA liberation from mitochondria: intracellular and extracellular release. In the extracellular release, cellular stress and necrosis are primary factors in the non-discriminant liberation of mitochondrial components such as mtDNA, *N*-formyl peptides, and cardiolipins, all of which could be mitochondrial DAMPs. Although it is easy to imagine the extracellular release of mitochondrial components in case of necrosis, it is unclear how extracellular mtDNA will activate intracellular PRR signalling factors such as TLR9, NLRP3, and the cGAS–STING DNA-sensing pathway. It is possible that internalization of mtDNA via endocytosis, transmembrane diffusion, phagocytosis, and receptor-mediated endocytosis contributes to the activation mechanism [73]; however, the precise mechanism remains to be determined. It is likely that mtDNA internalized through endocytosis can be detected by TLR9 on the membrane of the endosome in the endolysosomal compartment during autophagy. On the other hand, TLR9 has been detected on the surface of some types of cells, including resting B lymphocytes and peripheral blood monocytes, by using flow cytometry analysis, suggesting that direct activation of signalling could occur in those cell types [74–77].

While the accumulation of mitochondrial DAMPs including mtDNA has been shown to activate tissue-resident macrophages and favour tissue leukocyte infiltration [78], the actual mechanism of releasing mtDNA from non-necrotic cells remains unclear to date. As cell-free mtDNA is detected among the molecules released within exosomes [79], the exosomal release is proposed to be involved in the mechanism. In addition, it was reported that treatment of human neutrophils with ribonucleoprotein immune complexes induces mtROS, mtDNA oxidation, and translocation of mitochondria to the plasma membrane [80]. It was also shown that oxidized mtDNA is liberated to the extracellular space as a component of neutrophil extracellular traps [80].

Concerning the mechanism of intracellular release of mtDNA from mitochondria, the opening of mitochondrial permeability transition (MPT) pores plays an important role in mtDNA liberation through the mitochondrial membrane [81]. Inhibition of pore opening with cyclosporine A was reported to result in reduced mtDNA in the cytosol after stimulation with LPS and ATP [53]. Several reports suggested that mtDNA release is controlled by other MPT-associated regulatory proteins such as the voltage-dependent anion channel, hexokinase, Bax, and Bak [53,69,70,82]. The accumulated cytosolic mtDNA preferentially activates cGAS–STING signalling and type I IFN responses, without inflammasome activation, IL-1β production, or pro-inflammatory cytokine expression [69,70,72].

The degradation of extracellular mtDNA is important in inhibiting unnecessary inflammatory responses. In general, non-host DNA in the circulation is digested in part by circulating nucleases [83], and mtDNA may degrade in a similar mechanism. However, it is unclear whether nucleases actually digest mtDNA in the physiological condition, specifically in case of mtDNA existing in microvesicles such as exosomes, which can be protected from DNases. Intracellularly, DNaseII in autolysosomes has a central role in mtDNA degradation and mtDNA that escapes from the autophagic process stimulates inflammation [84].

## mtDNA and cardiac inflammation

While freely circulating mtDNA has been detected in plasma and serum in more than 60 studies on human diseases, there are few direct evidence that definitely show the significance of mtDNA in cardiac inflammation in the human heart. Circulating levels of mtDNA molecules increase along with aging and correlate with those of pro-inflammatory cytokines, including IL-6, TNF-α, and IL-1 receptor antagonist [85]. In addition, the concentration of circulating mtDNA is sufficient to activate cytokine production in monocytes, and mtDNA-induced inflammatory response can be involved in age-related cardiovascular diseases such as ischaemic heart diseases, heart failure, and atherosclerosis.

Mechanistically, multiple lines of evidence based on genetically engineered mouse models indicate the role of mtDNA-induced inflammation in cardiac pathology (Table 1). In addition to mtDNA sensors, the molecules related to mtDNA regulatory mechanisms potentially contribute to cardiac inflammation; however, their physiological roles have not been well defined. For instance, although overexpression of TFAM induces a protective effect in cardiac pathological models, the contributions of mtDNA regulation and cardiac inflammation in those models are not clear [86,87]. Similarly, deletion of CypD (cyclophilin D) leads to MPT inhibition and cardioprotection in an I/R (ischaemia/reperfusion) model [88,89]; however, mechanisms other than inhibition of necrotic cell death remain elusive, specifically with regard to mtDNA release via MPT pores and resultant inflammation. With regard to other mitochondrial DAMPs, the role of *N*-formyl peptides in the development of CVD remains totally unknown. In addition, the physiological role of cardiolipin as a DAMP has not been investigated in CVD, although its significance in mitochondrial function and morphology in the heart has been established [90].

**Table 1:** Cardiac phenotypes in genetically engineered mouse model related to mtDNA-induced inflammation

Genes	Type of genetically engineering	Experimental model	Cardiac phenotype	Cytokine or chemokine induction in heart
**mtDNA sensors**				
TLR9	KO	TAC	Increased cardiac function and survival	Decreased in IL-6 [84]
	KO	MI	No change	No change [97]
Inflammasomes				
NLRP3	KO	I/R	No change	Decreased in TNF-α [100]
	KO	I/R	Worsening	Decreased in TNF-α [99]
Caspase1	KO	I/R	Protection	Not described [98]
	TG (CA)	LPS challenge	Decreased cardiac function and survival	Increased in IL-1β [104]
ASC	KO	I/R	Protection	Decreased in TNF-α, IL-1β, IL-6 [98]
	KO	I/R	Worsening	Not significantly changed [99]
cGAS/STING pathway				
cGAS	KO	MI	Improved function and survival	Decreased in chemokine CXCL10 [96]
STING	KO	MI	No change	Decreased in chemokine CXCL10 [96]
IRF3	KO	MI	Improved function and survival	Decreased [96]
	KO	TAC	Exacerbation of cardiac hypertrophy	Not described [105]
	Cardio-specific TG	Aortic banding	Attenuation of cardiac hypertrophy	Not described [105]
**Genes potentially involved in mtDNA-induced inflammatory responses**				
TFAM	TG	MI	Improved function and heart failure	Not described [86]
	TG	Volume overload	Improved function	Not described [87]
CypD	KO	MI	Protection	Not described [119]
	KO	TAC	Worsening function	Not described [120]
	KO	I/R	Protection	Not described [88,89]
DNaseII	Cardio-specific KO	TAC	Worsening function	Increased in IL-6 [84]

Cardiac phonotypes characterized in genetically engineered mouse models are listed. Abbreviations: ASC: apoptosis-associated speck-like protein containing a caspase activation and recruitment domain; CA: constitutive active; cGAS: cyclic GMP–AMP synthetase; CXCL10: chemokine (C-X-C motif) ligand 10; CypD: cyclophilin D; IL: interleukin; I/R: ischemia/reperfusion; IRF3: interferon regulatory factor 3; KO: knockout mice; MI: myocardial infarction; NLRP3: nucleotide oligomerization domain-like receptor family pyrin domain containing 3; STING: stimulator of interferon genes; TAC: transverse aortic constriction; TFAM: transcription factor A, mitochondrial; TLR9: Toll-like receptor 9; TNF: tumour necrosis factor.

### Ischaemic heart diseases

Acute myocardial infarction is accompanied by massive cardiomyocyte necrosis and tissue inflammation. In multiple studies, it was reported that this extensive cardiomyocyte necrosis is also associated with elevated circulating mtDNA levels [91–93]. In addition, the increased level of mtDNA is reduced after reperfusion of the ischaemic myocardium, suggesting a close relation between myocardial damage and mtDNA [91,92]. In patients with diabetes mellitus, the occurrence of coronary artery disease (CAD) is related to higher mtDNA levels, suggesting the involvement of mtDNA-induced inflammatory responses in the development of CAD in those patients [94,95].

Modulation of mtDNA sensors can affect the cardiac phenotype in experimental models of ischaemic heart diseases. With regard to mtDNA sensors, King et al. [96] recently reported that mice genetically deficient in cGAS or STING exhibited impaired expression of IFN-stimulated genes, including *Cxcl10*. They also showed that interruption of IRF-3-dependent signalling leads to decreased expression of inflammatory cytokines and chemokines, attenuation of ventricular dilation, and improvement of cardiac function after myocardial infarction. Thus, cGAS-dependent signalling may play a vital role in mtDNA-induced inflammatory responses after myocardial infarction [96]. In contrast, TLR9 is not strongly involved in mtDNA-induced inflammation caused by cardiac ischaemic injury, based on experiments using *tlr9* null mice [97]. Multiple reports have investigated the pathological role of the NLRP3 inflammasome after myocardial infarction or I/R injury using ASC, caspase-1, or NLRP3 null mice [98–100]. However, conflicting results from those reports suggest the varied and complicated roles of the inflammasome in cardiac ischaemic injury.

### Heart failure

During heart failure, multiple endogenous DAMPs, including the intracellular S100 proteins, heat shock protein, HMGB1 (high-mobility group box 1), and mtDNA, are released and recognized by TLRs to induce an NF-κB-dependent inflammatory response [101]. Of note, extracellular mtDNA activates NF-κB through TLR9 in cardiomyocytes [102]. There are two types of heart failure based on systolic function: heart failure with preserved ejection fraction (HFpEF) and heart failure with reduced ejection fraction (HFrEF). Patients with HFpEF show symptoms of heart failure despite a lack of impaired cardiac systolic dysfunction. Currently, in the pathogenesis of HFpEF, a systemic pro-inflammatory state induced by microvascular endothelial cell inflammation is proposed to be a mechanism for HFpEF-specific phenotypes such as concentric cardiac remodelling and diastolic dysfunction [11].

Concerning HFrEF, direct cardiomyocyte damage or death leads to release of DAMPs, and it is considered that mitochondrial DAMPs including mtDNA cause cardiac inflammation, which contributes to the development of heart failure. However, there is no association between the severity of heart failure and the levels of serum mtDNA in patients with heart failure, although those patients show significantly higher levels of mtDNA than age- and sex-matched healthy controls [103].

Multiple studies have indicated the role of PRRs in heart failure by using genetically engineered mouse models. Deletion of *Tlr9* in mice results in the attenuation of inflammation and cardiac dysfunction in a pressure-overload-induced heart failure model. The involvement of mtDNA in cardiac inflammation is clearly demonstrated in this model, and a loss of sensing of mtDNA from damaged mitochondria in autolysosomes during mitophagy by TLR9 leads to the inactivation of the innate immunity in heart failure [84]. In regard to the role of inflammasomes in heart failure, targeted overexpression of a constitutively active form of NLRP3 failed to induce inflammasome formation in the heart [104]. However, after LPS stimulation, caspase-1 activation and cardiac dysfunction were observed in transgenic mice, whereas control mice showed no cardiac pathological phenotype. Further investigation of the molecular mechanism by which inflammasome activation leads to cardiac dysfunction is required. Finally, IRF3 is reported as a negative regulator of pathological hypertrophy based on gain- and loss-of-function study in genetically engineered mouse models [105]; however, the involvement of inflammation was not described.

### Atherosclerosis

Inflammation plays a central role in the development of atherosclerosis. In atherogenesis, metabolic stressors, such as fatty acids and cholesterol crystals, can activate the NLRP3 inflammasome, which is associated with mtDNA damage and stimulates inflammation. The activation of the NLRP inflammasome and the subsequent release of IL-1β in macrophages may promote atherosclerosis [61]. IL-1α also contributes to atherogenesis, as transplantation of IL-1α-deficient bone marrow into mice lacking the LDL receptor leads to reduction of atherosclerosis [106]. The stimulation of TLRs leads to up-regulation of type I IFNs including IFN-α and IFN-β. IFN-α causes death of vascular smooth muscle cells and IFN-β induces macrophage-endothelial cell adhesion and leukocyte recruitment to atherosclerotic lesions, which are essential steps in plaque formation [107]. Furthermore, increased type 1 IFN signalling has been confirmed in ruptured human plaques, indicating that mitochondrial DAMPs are important in the development of human atherosclerosis [108]. Indeed, mtDNA damage precedes and correlates with plaque development in human atherosclerosis [109,110]. In addition, mtDNA damage correlates with higher-risk lesions in patients [111]. Collectively, mitochondrial DAMPs are strongly involved in pro-atherogenic inflammatory signalling through the production of multiple IFNs and cytokines. Inflammasome activation is also involved in plaque formation [112]. Tumurkhuu et al. [112] reported that 8-oxoguanine glycosylase repairs oxidative DNA damage, including oxidized mtDNA, and prevents NLRP3 inflammasome activation in atherosclerotic plaques. Furthermore, Mao et al. [113] recently reported an important role of mitochondrial damage–cGAS–STING-dependent IRF3 signalling in metabolic stress-induced endothelial inflammation. Those evidence indicates that mtDNA and its sensors are strongly involved in the development of atherosclerotic diseases.

## Closing remarks

During sterile inflammation, a persistent inflammatory trigger raised in tissue-specific resident cells alarms circulating immune cells, which, in turn, respond by inducing a systemic response through the activation of mtDNA-induced inflammatory pathways. The release of cytokines, chemokines, nitric oxide, and ROS by inflammatory cells can elicit further mitochondrial damage, thereby developing a vicious circle, which reinforces the whole process leading to sterile inflammation. Similarly, these processes are considered to be involved in cardiac pathogenesis. Multiple reports have indicated that chronic inflammation plays a considerable role in heart failure. For instance, the levels of serum cytokines such as TNF-α, IL-6, and IL-1β are related to the severity of heart failure [18]. The increased levels of cytokines are attenuated, along with an improvement of prognosis after treatment with β-adrenergic blockers [114,115]. However, clinical trials targeting TNF-α signalling in patients with heart failure have demonstrated neutral results in terms of death and hospitalization [40], suggesting that the involvement of chronic inflammation in heart failure is not as simple as expected. In regard to acute inflammatory processes, a clinical trial using cyclosporine A, an MPT inhibitor that is expected to prevent necrotic cell death and mtDNA release from mitochondria, initially showed promising results in a pilot study [116]. However, subsequent multicentre trials demonstrated neutral results, possibly due to inappropriate doses or timing of administration [117,118]. On the basis of these clinical outcomes and the currently growing basic evidence on sterile inflammation, several remaining questions should be answered in order to develop novel therapeutics targeting cardiac inflammation. First, it should be determined which cells are suitable targets, among heart or systemic inflammatory cells, to improve outcome. There are too many potential sources that release triggering cytokines in the heart, and the initial target for inhibition should be determined. Second, the corresponding sensing system with mtDNA for suitable cell targeting needs to be defined. Third, tools for inhibiting mtDNA-induced inflammatory response in specific cell types need to be established, and the lack of a systemic inhibitory effect should be resolved by finding specific molecules or by developing drug delivery systems that can selectively transfer the drugs to the targeted cells. For instance, cyclosporine A, which is clinically used as an immunosuppressant, is not suitable for chronic use, as it compromises the essential immune reaction.

Finding precise answers to the above-mentioned issues will lead to the development of novel therapies targeting cardiac inflammation in patients with cardiovascular diseases, to improve prognosis.

## Abbreviations

ASC: apoptosis-associated speck-like protein containing a caspase activation and a recruitment domain
CAD: coronary artery disease
cGAS: cyclic GMP–AMP synthetase
CXCL10: chemokine (C-X-C motif) ligand 10
CypD: cyclophilin D
DAMP: damage-associated molecular pattern
HFpEF: heart failure with preserved ejection fraction
HFrEF: heart failure with reduced ejection fraction
IFN: interferon
IL: interleukin
I/R: ischemia/reperfusion
IRF: interferon regulatory factor
LPS: lipopolysaccharide
MPT: mitochondrial permeability transition
mtDNA: mitochondrial DNA
mtROS: mitochondrial ROS
NET: neutrophil extracellular trap
NF-κB: nuclear factor kappa B
NLR: nucleotide oligomerization domain-like receptor
NLRP3: NLR family pyrin domain containing 3
PAMP: pathogen-associated molecular pattern
PRR: pattern recognition receptor
ROS: reactive oxygen species
STING: stimulator of interferon genes
TFAM: transcription factor A, mitochondrial
TGF: transforming growth factor
TLR: Toll-like receptor
TNF: tumour necrosis factor
